# The Faith Healer’s Idolatry

**Published:** 1899-12

**Authors:** 


### Selection.

### THE FAITH HEALER'S IDOLATRY.

[*American Medical Quarterly*, September.]

THE somewhat startling proportions into which the various forms of belief in the efficacy of prayer to heal disease has grown, justifies a full and unimpassioned discussion of the subject in the medical journals and magazines. While the healer confined his work to those persons who had reached the age of discretion and limited their pretended ministrations to diseases of benignity, or to affections of the imagination, no great harm could result. But, emboldened by the pretense of success on the one hand, and by the indifference of the general public on the other, he is stimulated to invade fields that make him a dangerous member of the body politic, if not a downright outlaw.

He denies the contagiousness of disease, sends infected children to the public schools, and spreads contagion with a recklessness that brings dismay to the intelligent and thoughtful citizen. He says diphtheria is not fraught with danger because it is not susceptible of transmission from person to person; he treats scarlet fever in the same manner that he would a stone bruise, and even denies that there is such a malady as smallpox. An epidemic of cholera would be a camp-meeting revel and the bubonic plague a carnival of faith-curing glory, in which he would display the wonderful healing powers of his divine gift. A touch, a blessing or an invocation, and lo! they vanish into thin air, into the realms of immunity, where disease is unknown and where suffering cannot enter!

Nay, more; cancer, consumption, syphilis and gonorrhea cannot exist, because there is neither disease, curable or incurable, nor contagion, infection or heredity.

But the faith healer does not stop at disease: he offers his ministrations at childbirth and would pray in the midst of the alarming flooding of placenta previa or post-partum hemorrhage with the same assurance and confidence of success that the prestidigitateur boils eggs in a new silk hat, borrowed from an auditor, to whom he promises it back unsullied, or exhibits the vanishing lady to a surprised assembly. A ruptured uterus, puerperal eclampsia, and a two and a half inch conjugate hath no terrors for him, while asphyxia and ophthalmia neonatorum are purely fictions of an unbelieving imagination.

Our progressive and aggressive "healer" even would invade the domain of surgery and undertake the care of a broken leg or a wound of the intestines, praying the fragments into aposition, or the several tissues into continuity. But we need not call attention further to the absurdities, inconsistencies or impossibilities which this strange sect would practise. At first glance these claims may seem apocryphal to the reader. We beg to assure the incredulous that they are not exaggerated. We have heard some or all of these claims made, in reality or impliedly, in a public audience chamber.

During the past few months the daily newspapers have contained many accounts of the workings of these singular people, and the victims of their practices are becoming so numerous as to make the record positively startling. Especially is this the case among children or young persons below the age of responsibility. Misguided parents have assumed to deny them the aid of lawful medical attendants, and they have been allowed to die neglected and, in many instances, in the agony of suffering. All this, too, in the closing year of the XIXth. Century, admittedly the most enlightened period of the world.

The dangers to the public weal from the unrestricted practice of these methods, and the propagation of these dogmas of faith cure are apparent, and sooner or later must arouse an indignation that will be as swift in judgment by contrast as it has been slow to anger.

Meanwhile the difficulties in correcting the evils are many, and are not easily overcome. Prosecuting attorneys are often tainted with the belief or have been told that some of their constituents have been cured through the ministrations of the accused "after the doctors had given up" the patient. Grand jurors, too, arise in their places, pending the consideration of a proposed indictment, and tell of the wonderful cures that have been performed within their own knowledge by the prisoner. Lawyers, also, of greater or less distinction, can always be found to espouse the cause of a healer in the toils, and cunningly distort the facts to prove that these people do not practice medicine because they prescribe no drugs.

There is another great danger that threatens the miscarriage of justice in these cases. Judges are likely to be chosen from among these same lawyers, hence the bench may become a propagandist of this monstrous faith.

In a former issue the *Quarterly* took the ground that the existing statutes are all sufficient to meet the demands of justice in these cases. We still think so; but it, nevertheless, remains for the public to become well informed as to the dangers that lurk in the midst of indifference, and to demand an obedience to the laws that relate to the practice of medicine in the several states. They are framed in the interests of the people for their protection against ignorance and deception. Let them be obeyed !

